# Genomic Organization and Expression of Iron Metabolism Genes in the Emerging Pathogenic Mold *Scedosporium apiospermum*

**DOI:** 10.3389/fmicb.2018.00827

**Published:** 2018-04-26

**Authors:** Yohann Le Govic, Nicolas Papon, Solène Le Gal, Bénédicte Lelièvre, Jean-Philippe Bouchara, Patrick Vandeputte

**Affiliations:** ^1^Groupe d'Etude des Interactions Hôte-Pathogène (EA 3142), SFR ICAT 4208, UNIV Angers, UNIV Brest, Angers, France; ^2^Laboratoire de Parasitologie-Mycologie, Centre Hospitalier Universitaire, Angers, France; ^3^Groupe d'Etude des Interactions Hôte-Pathogène (EA 3142), SFR ICAT 4208, UNIV Angers, UNIV Brest, Brest, France; ^4^Laboratoire de Parasitologie-Mycologie, Centre Hospitalier Universitaire, Brest, France; ^5^Laboratoire de Pharmacologie-Toxicologie, Centre Hospitalier Universitaire, Angers, France

**Keywords:** *Scedosporium*, genome mining, iron, siderophore, gene expression

## Abstract

The ubiquitous mold *Scedosporium apiospermum* is increasingly recognized as an emerging pathogen, especially among patients with underlying disorders such as immunodeficiency or cystic fibrosis (CF). Indeed, it ranks the second among the filamentous fungi colonizing the respiratory tract of CF patients. However, our knowledge about virulence factors of this fungus is still limited. The role of iron-uptake systems may be critical for establishment of *Scedosporium* infections, notably in the iron-rich environment of the CF lung. Two main strategies are employed by fungi to efficiently acquire iron from their host or from their ecological niche: siderophore production and reductive iron assimilation (RIA) systems. The aim of this study was to assess the existence of orthologous genes involved in iron metabolism in the recently sequenced genome of *S. apiospermum*. At first, a tBLASTn analysis using *A. fumigatus* iron-related proteins as query revealed orthologs of almost all relevant loci in the *S. apiospermum* genome. Whereas the genes putatively involved in RIA were randomly distributed, siderophore biosynthesis and transport genes were organized in two clusters, each containing a non-ribosomal peptide synthetase (NRPS) whose orthologs in *A. fumigatus* have been described to catalyze hydroxamate siderophore synthesis. Nevertheless, comparative genomic analysis of siderophore-related clusters showed greater similarity between *S. apiospermum* and phylogenetically close molds than with *Aspergillus* species. The expression level of these genes was then evaluated by exposing conidia to iron starvation and iron excess. The expression of several orthologs of *A. fumigatus* genes involved in siderophore-based iron uptake or RIA was significantly induced during iron starvation, and conversely repressed in iron excess conditions. Altogether, these results indicate that *S. apiospermum* possesses the genetic information required for efficient and competitive iron uptake. They also suggest an important role of the siderophore production system in iron uptake by *S. apiospermum*.

## Introduction

Iron is the fourth most common element found on the Earth's crust (Frey and Reed, 2012). However, in spite of its abundance, iron is fairly accessible to living organisms as a result of its very limited solubility under aerobic conditions. Iron is mainly encountered in two relatively stable oxidation states, ferrous (Fe(II) or Fe^2+^) and ferric (Fe(III) or Fe^3+^). Due to the reversible switching between the Fe^2+^ and Fe^3+^ species and its ability to form coordination complexes with organic ligands, iron plays a critical role in numerous biochemical processes including oxidative phosphorylation, DNA replication, and biosynthesis of small molecules such as lipids, amino acids, and sterols (Philpott, 2006). On other hand, iron excess can be harmful to the cell owing to its capacity to catalyze the formation of reactive oxygen species (ROS) and to initiate lipid peroxidation (Halliwell and Gutteridge, 1984). Therefore, microbes have evolved sophisticated systems to overcome suboptimal iron availability and meanwhile to prevent iron overload toxicity.

Four distinct mechanisms of iron acquisition have been described in fungi: (i) heme uptake and degradation, (ii) low-affinity ferrous iron uptake, which occurs through relatively non-specific divalent cation transporters, (iii) reductive iron assimilation (RIA), employing a high-affinity uptake system in which ferrous iron is first oxidized by a multicopper-ferroxidase before being transferred to the cytosol via a specific Fe(III)-permease, and (iv) siderophore-mediated iron uptake (Haas, 2014). Siderophores are amongst the strongest natural Fe(III)-chelating products. The majority of fungal siderophores belongs to the hydroxamate class and can be divided into four structural families: rhodotorulic acid, fusarinines, coprogens, and ferrichromes. The hydroxamate functional group is synthesized from L-ornithine, a non-proteinogenic amino acid that is produced either in mitochondria from L-glutamate or in the cytosol through hydrolysis of L-arginine (Schafferer et al., 2015). The first key enzyme of the hydroxamate biosynthetic pathway is the L-ornithine-*N*^5^-monooxygenase SidA, which catalyzes *N*^5^-hydroxylation of L-ornithine (Eisendle et al., 2003). The hydroxamate motif is then formed by *N*^5^-acylation of *N*^5^-hydroxy-L-ornithine by *N*^5^-transacylases. Here, the pathway splits as different acyl groups can be attached to hydroxyornithine, defining the nature of the siderophore produced. In *Aspergillus fumigatus*, this step is mediated by two transacetylases: SidL, which adds an acetyl group in ferrichrome-type siderophores (Blatzer et al., 2011c), and SidF, which adds an anhydromevalonyl group in siderophores of the coprogen and fusarinine families (Schrettl et al., 2007). Anhydromevalonyl-CoA is obtained from mevalonate by consecutive CoA-ligation and dehydration catalyzed by the peroxisomal enzymes SidI and SidH, respectively (Yasmin et al., 2012; Gründlinger et al., 2013). The ultimate step consists in the covalent linkage of the *N*^5^-acyl-*N*^5^-hydroxy-L-ornithine groups, and is orchestrated by non-ribosomal peptide synthetases (NRPSs). After being activated by a 4′-phosphopantetheinyltransferase protein (NpgA/PptA), the NRPSs SidC and SidD achieve assembly of intra- and extracellular siderophores in *A. fumigatus*, respectively (Schrettl et al., 2007). Excreted siderophores bind Fe(III) ions to form ferrisiderophores chelates, which are then imported into the cell through specific plasma membrane-localized transporters termed “siderophore-iron transporters” (SITs). At least two types of SITs have been identified in the aspergilli, including *Aspergillus* SitT and MirA-D proteins, which belong to the ATP-binding cassette and major facilitator superfamilies, respectively (Haas et al., 2003; Schrettl et al., 2008, 2010). Once entered into the cytoplasm, iron is released from siderophores and finally becomes available for various cellular processes.

In addition to the non-reductive siderophore-mediated iron acquisition, pathogenic fungi developed a reductive, non-chelating high affinity iron uptake system called “reductive iron assimilation” (RIA). RIA necessitates the reduction by a ferric reductase (Fre family) of highly insoluble ferric iron to more soluble and bioavailable ferrous iron, combined with a specific transport system composed of a multicopper ferroxidase (Fet family) associated with a ferric permease (Ftr family) (Kosman, 2013). The Fet and Ftr proteins are inextricably linked together since they are assembled into a stable complex prior to plasma membrane trafficking (Stearman et al., 1996). Indeed, the Fet-mediated Fe(II) oxidation step is mandatory to the permeation step, i.e., the Ftr channel only accepts Fe(III) generated by the coupling Fet protein (Wang et al., 2003). Unlike *Cryptococcus neoformans* and *Candida albicans* ferric permeases, *A. fumigatus* FtrA has been demonstrated to be dispensable for fungal virulence, in the presence of a functional siderophore iron uptake system (Ramanan and Wang, 2000; Schrettl et al., 2004; Jung et al., 2008). Furthermore, ferric reductases play an important role in the removal of iron from siderophores (Yun et al., 2001) or from host iron sources, such as heme and transferrin (Knight et al., 2005; Saikia et al., 2014).

In *A. fumigatus*, optimal iron balance is maintained by two central regulatory proteins, which are interconnected in a negative feedback loop: the GATA-transcription factor SreA and the bZIP-transcription factor HapX (Haas, 2014). During iron starvation, HapX represses iron-consuming pathways (e.g., heme biosynthesis and respiration) and activates siderophore production through interaction with the CCAAT-binding complex (CBC). On the other hand, during iron sufficiency, SreA down-regulates both RIA and siderophore uptake systems via binding to the consensus DNA sequence ATCWGATAA. The disruption of *hapX*, but not of *sreA*, was shown to impair virulence of *A. fumigatus* in murine models of invasive aspergillosis (Schrettl et al., 2008, 2010). These observations highlight the need to adapt to iron limitation for establishing fungal infection, which is consistent with the fact that intra- and extracellular siderophores play a pivotal role in *A. fumigatus* virulence (Schrettl et al., 2007).

*Scedosporium apiospermum* is a ubiquitous fungus capable of causing a wide range of infections in human (Cortez et al., 2008). Despite numerous studies showing an increasing health threat, especially among patients with underlying conditions (e.g., immunodeficiency or cystic fibrosis) (Walsh and Groll, 1999; Guarro et al., 2006; Lamaris et al., 2006; Pihet et al., 2009; Douglas et al., 2016; Koehler et al., 2016; Chen et al., 2017), little is known about virulence factors enabling the fungus to produce acute or chronic infections. Moreover, *Scedosporium* infections are extremely difficult to treat due to the high level of intrinsic resistance to many, if not all, of current antifungals (Cortez et al., 2008). To gain insight into the pathogenic and drug resistance mechanisms of this fungus, the genome of a clinical isolate of *S. apiospermum* was fully sequenced in 2014 (Vandeputte et al., 2014). Here, we describe the first genomic and transcriptional analysis focusing on genes related to iron metabolism in *S. apiospermum*.

## Materials and methods

### Strain and culture conditions

*Scedosporium apiospermum* (*S. apiospermum*; taxid:563466) whole-genome sequenced strain IHEM 14462, originally isolated from a sputum sample from a cystic fibrosis patient (Vandeputte et al., 2014), was grown on Potato Dextrose Agar (Conda, Madrid, Spain) plates at 37°C for 9 days to induce sporulation. Conidia were harvested from colonies by aseptically scraping the plates using 1X TE buffer (10 mM Tris(hydroxymethyl)aminomethane, HCl pH 7.5, 1 mM EDTA NaOH pH 8) and passing through Miracloth® mesh filter (Merck, Darmstad, Germany) to remove the mycelia. The filtrate was centrifuged (4,600 rpm, 5 min) and pelleted conidia were resuspended in 1X TE buffer. Conidia were numbered with a hemocytometer and a total of 10^7^ conidia were inoculated into 100-ml flasks containing 25 ml of YEPD medium (containing per liter: 5 g yeast extract, 10 g peptone, 20 g dextrose, and 0.5 g chloramphenicol). Cultures were incubated for 48 h at 37°C with agitation (120 rpm). Iron excess was obtained by supplementing YEPD medium with 20 μM of either free (FeSO_4_ or FeCl_3_) or transferrin-bound iron (holotransferrin, Thermo Fisher, Karlsruhe, Germany). Iron-depleted conditions were obtained by adding 200 μM bathophenanthroline disulfonate (BPS, Sigma-Aldrich, Saint-Quentin Fallavier, France) in YEPD medium.

### Genome mining

Identification of *S. apiospermum* genes potentially involved in iron metabolism was performed as described by Haas (Haas, 2012), searching for orthologs of *A. fumigatus* strain Af293 (*A. fumigatus*; taxid:330879) iron-related proteins through tBLASTn analysis (https://blast.ncbi.nlm.nih.gov/Blast.cgi) against *S. apiospermum* genome. Only results corresponding to strain IHEM 14462 with an *e*-value < 1e-15 on at least 40% of the query sequence were considered. Organization into clusters of the genes found in *S. apiospermum* genome was further compared with those identified in the genomes of *A. fumigatus* strain Af293 (Nierman et al., 2005), *A. nidulans* strain FGSC A4 (taxid:227321) (Galagan et al., 2005), *A. niger* strain CBS 513.88 (taxid:425011) (Pel et al., 2007), *Colletotrichum higginsianum* strain IMI 349063 (taxid:759273) (O'Connell et al., 2012) and *Trichoderma reesei* strain QM6a (taxid:431241) (Martinez et al., 2008). Searching for putative binding sites of the transcription factor HapX (Hortschansky et al., 2007) was performed within the 2 kb upstream region of each gene putatively involved in RIA and siderophore metabolism by using the MEME Suite's FIMO (Grant et al., 2011).

### RNA isolation, retrotranscription and real-time quantitative PCR

Fungal cells from triplicate cultures in standard, iron-overloaded and iron-depleted conditions were harvested at 48 h and ground in liquid nitrogen with a mortar and pestle. Total RNA was recovered by processing the fungal powder with the NucleoSpin® RNA Plant kit (Macherey-Nagel, Düren, Germany), according to the manufacturer's instructions. All RNA samples (5 μg) were treated with 2 U of RNase-free DNase I (Ambion^TM^ Life Technologies, Carlsbad, CA), according to the protocol supplied by the manufacturer. Complementary DNA were synthesized from 500 ng total RNA using SuperScript IV reverse transcriptase (200 U; Invitrogen Life Technologies, Carlsbad, CA) in the presence of oligo-d(T) primer (2.5 μM), deoxyribonucleoside triphosphates (0.5 mM each), dithiotreitol (5 mM), and RNase inhibitor (2 U). Thereafter, cDNA were 20-fold diluted and used as template for real-time quantitative PCR (qPCR). Each reaction (12.5 μl final volume) contained Fast SYBR® Green PCR Master Mix (Applied Biosystems, Foster City, CA), 200 nM of each primer (Integrated DNA Technologies Inc., Leuven, Belgium), and 1 μl of diluted cDNA. Primers used to perform qPCR experiments are compiled in Supplementary Table S1. qPCR reactions were carried out on a StepOnePlus^TM^ thermocycler (Applied Biosystems) with the following thermal profile: 95°C for 2 min, 40 cycles of 95°C for 3 s, 60°C for 30 s. Melting curve analysis was performed immediately after the amplification procedure as follows: 95°C for 15 s, and stepwise annealing from 60 to 94.9°C with 0.3°C increments. For each gene, fold changes relative to standard condition (i.e., YEPD medium) were calculated in each condition using the delta-delta Ct method and *ubcB* and *sarA* genes as endogenous controls (Llanos et al., 2015). For each data point, three biological replicates and two technical replicates were performed, and the variation in expression of a given gene was considered significant if the log2 fold change ± standard deviation was > 1 or < −1.

## Results

### Genome mining for iron homeostasis in *Scedosporium apiospermum*

**Figure 1:**
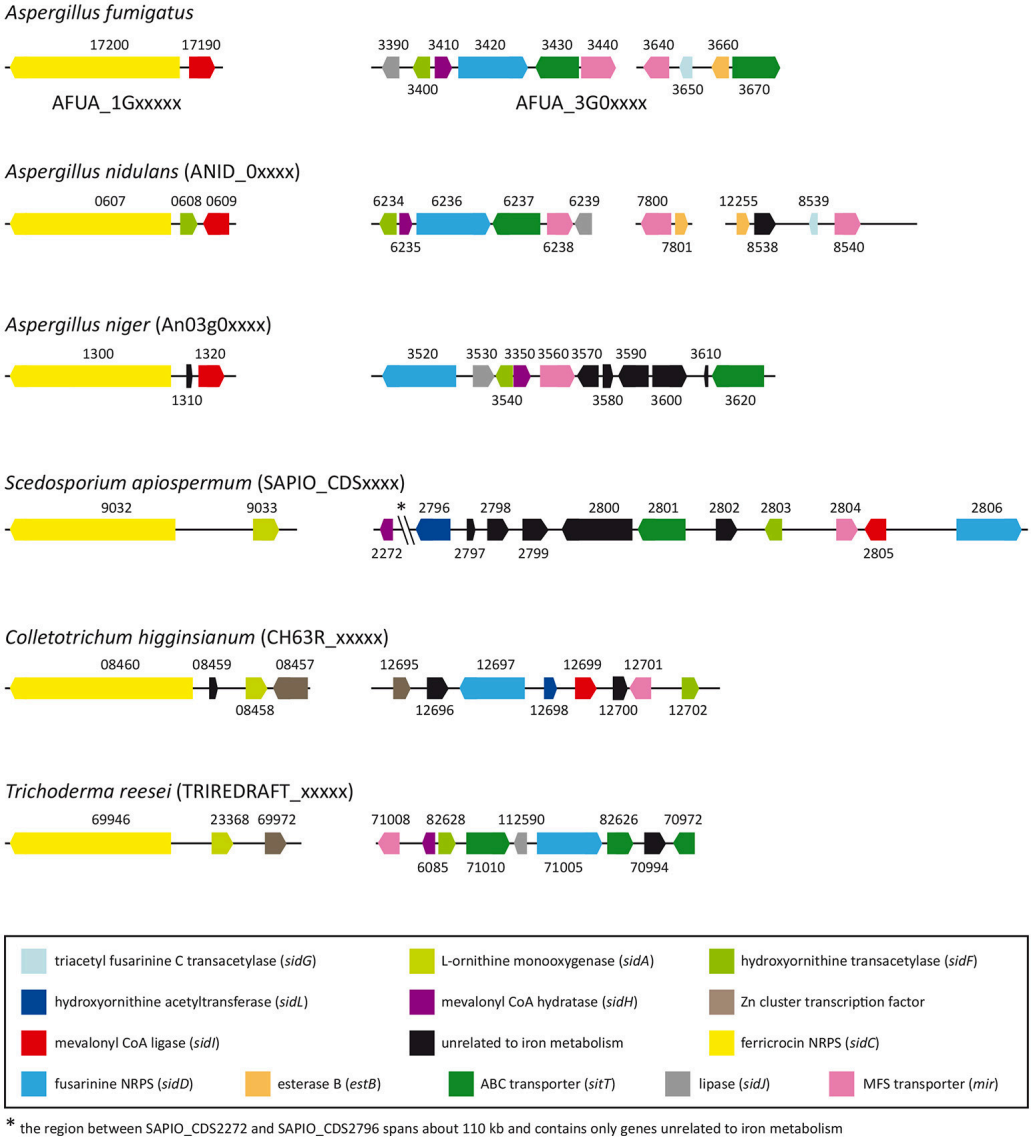
Distribution of siderophore biosynthetic and transporter genes in *S. apiospermum* IHEM 14462, *A. fumigatus* Af293, *A. nidulans* FGSC A4, *A. niger* CBS 513.88, *C. higginsianum* IMI 349063, and *T. reesei* QM6a genomes. Most of the siderophore-related genes are clustered within all the six filamentous fungi, but the content of these clusters varies from one species to another. Of note, gene organization in *S. apiospermum* is closer to that observed in *C. higginsianum* and *T. reesei*. Orthologous genes, taking *A. fumigatus* as reference, are identically colored. (adapted from Franken et al., 2014).

Computational identification of genes putatively involved in iron metabolism in *S. apiospermum* was performed through a tBLASTn analysis, using *A. fumigatus* Af293 iron-related proteins as query (Haas, 2012). This strategy allowed to find orthologs of all genes involved in iron acquisition and storage (Table 1), with the exception of *srbA*, which encodes a regulatory protein that activates iron uptake during iron deprivation (Blatzer et al., 2011a), *sidG*, which encodes a protein that catalyzes fusarinine C esterification, and *estB* and *sidJ*, which both encode proteins involved in triacetylfusarinine C saponification (Kragl et al., 2007; Schrettl et al., 2007). Furthermore, this analysis revealed that *S. apiospermum* genome contains *two* putative gene clusters harboring an iron-related NRPS as the core member (Figure 1). Indeed, the closest orthologs of these NRPS genes, *sidC* and *sidD*, are known or presumed to be involved in siderophore synthesis in *A. fumigatus* (Haas, 2012)*, A. nidulans* (von Döhren, 2009), and *A. niger* (Franken et al., 2014). In *S. apiospermum*, the *sidC*-related cluster contains an ortholog of *sidA*, which controls the initiation step of fungal hydroxamate siderophore biosynthesis (Eisendle et al., 2003). The second cluster is a combination made up of six genes putatively involved in siderophore production (i.e., *sidD, sidF, sidI*, and *sidL* orthologs) and transport (i.e., one *sitT* and one *mir* orthologs). The *sidH* ortholog is located in a different region of the genome together with a putative MFS transporter gene (CDS2271), and is separated from the *sidD* cluster by 110 kb. Data mining also revealed the existence of 7 other *mir* orthologs randomly distributed within *S. apiospermum* genome.

**Table 1:** Results of tBLASTn analysis of the genes putatively involved in iron metabolism in *S. apiospermum* against *A. fumigatus* Af293 (taxid: 330879).

**Protein**	**Function**	***A. fumigatus* coding sequence**	***S. apiospermum* ortholog (E-value/max identity compared with *A. fumigatus* protein)**	**Query cover(%)**	***S. apiospermum* encoded protein (Genbank accession number)**
**SIDEROPHORE BIOSYNTHESIS**					
SidA	L-ornithine-*N*^5^-monooxygenase	AFUA_2G07680	SAPIO_CDS9033 (3e-112/52%)	90	KEZ40036.1
SidC	NRPS ferricrocin	AFUA_1G17200	SAPIO_CDS9032 (0.0/26%)	94	KEZ40035.1
SidD	NRPS fusarinine C	AFUA_3G03420	SAPIO_CDS2806 (0.0/44%)	88	NW_015971788.1^*^
SidF	Hydroxyornithine transacylase	AFUA_3G03400	SAPIO_CDS2803 (6e-125/47%)	88	NW_015971788.1^*^
SidG	Transacetylase	AFUA_3G03650	Ø		
SidH	Mevalonyl-CoA hydratase	AFUA_3G03410	SAPIO_CDS2272 (4e-80/48%)	97	NW_015971787.1^*^
SidI	Mevalonyl-CoA ligase	AFUA_1G17190	SAPIO_CDS2805 (2e-143/64%)	96	KEZ44717.1
SidL	Transacetylase	AFUA_1G04450	SAPIO_CDS2796 (7e-120/42%)	100	KEZ44711.1
EstB	Triacetylfusarinine C esterase	AFUA_3G03660	Ø		
SidJ	Lipase/Esterase	AFUA_3G03390	Ø		
PptA	Phosphopantetheinyl transferase	AFUA_2G08590	SAPIO_CDS5197 (3e-26/31%)	74	KEZ42787.1
AgaA	Arginase	AFUA_3G11430	SAPIO_CDS10183 (5e-153/64%)	98	KEZ38874.1
AmcA	Mitochondrial ornithine carrier protein	AFUA_8G02760	SAPIO_CDS3378 (6e-87/48%)	92	KEZ44391.1
**SIDEROPHORE TRANSPORT SYSTEMS**					
MirB	MFS transporter	AFUA_3G03640	SAPIO_CDS2478 (1e-153/57%)	88	KEZ45056.1
			SAPIO_CDS2804 (4e-105/37%)	87	NW_015971788.1^*^
			SAPIO_CDS9285 (2e-87/36%)	83	KEZ40224.1
			SAPIO_CDS4564 (4e-84/35%)	83	KEZ43394.1
			SAPIO_CDS4736 (2e-51/32%)	85	KEZ43292.1
			SAPIO_CDS5249 (2e-46/26%)	87	NW_015971799.1^*^
			SAPIO_CDS6391 (6e-37/32%)	46	KEZ41999.1
			SAPIO_CDS1833 (9e-35/30%)	62	NW_015971778.1^*^
MirC	MFS transporter	AFUA_2G05730	SAPIO_CDS2804 (2e-52/28%)	82	NW_015971788.1^*^
			SAPIO_CDS5249 (1e-48/27%)	82	NW_015971799.1^*^
			SAPIO_CDS4564 (6e-48/28%)	79	KEZ43394.1
			SAPIO_CDS9285 (1e-46/26%)	93	KEZ40224.1
			SAPIO_CDS1833 (9e-39/28%)	64	NW_015971778.1^*^
			SAPIO_CDS6391 (2e-38/30%)	63	KEZ41999.1
			SAPIO_CDS2478 (4e-22/28%)	78	KEZ45056.1
			SAPIO_CDS4736 (1e-19/27%)	41	KEZ43292.1
MirD	MFS transporter	AFUA_3G03440	SAPIO_CDS2478 (2e-137/46%)	88	KEZ45056.1
			SAPIO_CDS2804 (2e-93/33%)	92	NW_015971788.1^*^
			SAPIO_CDS9285 (4e-88/32%)	96	KEZ40224.1
			SAPIO_CDS4564 (2e-80/35%)	85	KEZ43394.1
			SAPIO_CDS4736 (3e-37/32%)	90	KEZ43292.1
			SAPIO_CDS5249 (6e-34/24%)	94	NW_015971799.1^*^
			SAPIO_CDS6391 (2e-24/29%)	53	KEZ41999.1
			SAPIO_CDS1833 (2e-22/24%)	61	NW_015971778.1^*^
SitT	ABC transporter	AFUA_3G03430	SAPIO_CDS2801 (0.0/48%)	99	KEZ44715.1
CccA	Vacuolar iron transporter	AFUA_4G12530	SAPIO_CDS5446 (1e-57/43%)	68	KEZ42991.1
**REGULATORY PROTEINS**					
AcuM	Zn2Cys6 transcription factor	AFUA_2G12330	SAPIO_CDS0915 (7e-79/48%)	65	KEZ46068.1
MpkA	MAP kinase A	AFUA_4G13720	SAPIO_CDS2689 (1e-162/73%)	99	KEZ45223.1
PacC	Cys2His2 transcription factor	AFUA_3G11970	SAPIO_CDS0213 (5e-42/66%)	69	KEZ46879.1
SreA	ZnF_GATA transcription factor	AFUA_5G11260	SAPIO_CDS7310 (1e-34/39%)	40	KEZ41223.1
SrbA	bHLH transcription factor	AFUA_2G01260	Ø		
HapX	bZip transcription factor	AFUA_5G03920	SAPIO_CDS9738 (8e-22/30%)	49	NW_015971844.1^*^
**REDUCTIVE IRON ASSIMILATION**					
FreB	Ferric reductase	AFUA_1G17270	SAPIO_CDS2383 (5e-67/38%)	75	KEZ44995.1
			SAPIO_CDS1476 (1e-46/28%)	69	KEZ45701.1
			SAPIO_CDS9014 (2e-39/30%)	48	KEZ40025.1
			SAPIO_CDS10508 (4e-37/23%)	69	KEZ39117.1
			SAPIO_CDS10060 (4e-30/24%)	70	NW_015971855.1^*^
			SAPIO_CDS9433 (1e-28/26%)	56	KEZ39544.1
			SAPIO_CDS5404 (7e-24/24%)	69	KEZ42955.1
			SAPIO_CDS6952 (2e-17/26%)	49	NW_015971810.1^*^
			SAPIO_CDS10726 (2e-17/23%)	41	KEZ38703.1
FetC	Multicopper ferroxidase	AFUA_5G03790	SAPIO_CDS0314 (2e-103/55%)	96	KEZ46527.1
			SAPIO_CDS8659 (1e-62/51%)	87	KEZ40718.1
			SAPIO_CDS0322^#^ (0.0/54%)^$^		KEZ46534.1
FtrA	Iron permease	AFUA_5G03800	SAPIO_CDS0321 (3e-95/52%)	94	KEZ46533.1
			SAPIO_CDS0315^#^ (2e-107/49%)^$^		KEZ46528.1

* *Accession number of the contig since the corresponding CDSs are considered as pseudogenes in the draft genome sequence of S. apiospermum*;

Ø *, not present or score below thresholds (e-value: 1e-15, query cover: 40%)*;

# *, putative orthologs detected through blastP analysis against fungi (taxid:4751)*;

$ *, best scores obtained with blastP against Aspergillus fumigatus Af293*.

**Figure 2:**
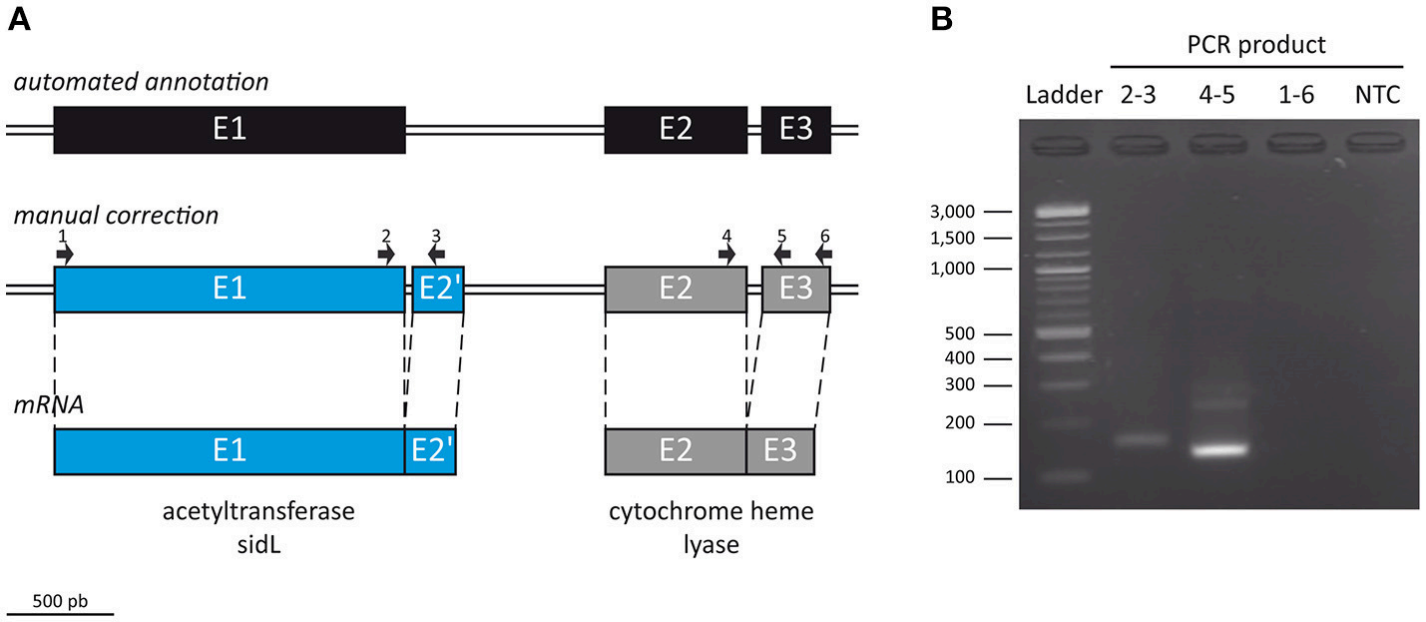
Manual correction of ORF SAPIO_CDS2796 annotation. **(A)** To further refine the automated annotation performed on the genome of *S. apiospermum* IHEM 14462 (top), the size of the mRNA transcribed from CDS2796 was assessed by RT-PCR using 3 pairs of primers (middle): one pair spanning the newly discovered intron (2–3), another covering the last intron (4–5), and a third pair covering CDS2796 from start to stop codons as automatically annotated (1–6). **(B)** Gel electrophoresis analysis of the PCR products. The first primer pair (2–3) amplified an expected 123 bp-fragment, which confirms the existence of an intron at the end of E1. The second couple (4–5) also amplified a fragment of the expected size. On the opposite, the last pair of primers (1–6) gave no amplification, further confirming that the transcription of CDS2796 produces two mRNAs, the upstream one corresponding to a *sidL* ortholog (**A**, bottom). NTC, no-template control.

Of note, we found that the *S. apiospermum sidL* gene was not correctly annotated (Figure 2). Indeed, this coding region (CDS2796) is made of three exons – E1 (1,469 bp), E2 (600 bp), and E3 (277 bp) – and two introns – I1 (783 bp) and I2 (73 bp) – while in *A. fumigatus* and *A. nidulans, sidL* gene contains only 2 exons (~1,400 and 120 bp, respectively) separated by a ~50 bp intron. In other words, the *S. apiospermum sidL* ORF is twice longer than its *Aspergillus* orthologs, partly due to a long first intron. Interestingly, a Pfam analysis (http://pfam.xfam.org/) of the deduced protein sequence identified two conserved motifs in CDS2796: an acetyltransferase (GNAT) domain as expected for *sidL*, but also a cytochrome heme lyase domain. This discrepancy led us to refine the analysis of CDS2796 sequence which revealed the presence of previously undetected exon-intron boundaries, one located at the 3′ end of E1, and another 96 bp away (i.e., inside I1), from which the transcription of a 120 bp supplementary exon occurs. The size of the mRNA transcribed from CDS2796 was further confirmed experimentally by designing 3 pairs of primers (Figure 2A). The first pair spans the newly discovered intron, and a PCR performed on cDNA with these primers amplified an expected 123 bp-fragment, which confirms the existence of this predicted intron (Figure 2B). The second pair of primers was designed to cover the last intron, and also amplified a fragment of the expected size. On the opposite, the third pair of primers, covering CDS2796 from start to stop codons as automatically annotated, gave no amplification, further confirming that the transcription of CDS2796 produces two mRNAs, the most upstream corresponding to a *sidL* ortholog, in agreement with the sequence of this protein in *Aspergillus* species. The annotation of the contig containing the *S. apiospermum sidL* gene (GenBank accession number NW_015971788.1) has been updated accordingly.

Aside from siderophore-mediated iron acquisition gene battery, the tBLASTn analysis allowed the detection of several genes putatively involved in RIA. Indeed, two *A. fumigatus fetC* and one *ftrA* orthologs were identified in *S. apiospermum*. The *fetC* ortholog displaying the lowest similarity (CDS8659) with *A. fumigatus* has no iron-related genes in its vicinity; however, this ORF is clustered with genes involved in melanin biosynthesis, which requires the action of multicopper oxidases belonging to the laccases subfamily. These enzymes catalyze the oxidation of phenolic compounds that simultaneously converts Fe(III) to Fe(II), suggesting rather a role for CDS8659 in melanin production through a laccase (Fe(III)-reducing) activity. The two others *fetC/ftrA* putative orthologs (CDS0314 and CDS0321, respectively) are separated by about 28 kb. However, in fungi, these proteins are classically encoded by paired consecutive genes oriented on the opposite strand one from another (Kensche et al., 2008). The genes neighboring CDS0314 and CDS0321 therefore were analyzed through a BLASTp analysis against fungal genomic resources (taxid:4751). This allowed the identification of CDS0322 as a *fetC* ortholog, paired with the *ftrA* ortholog CDS0321, and of CDS0315 as an *ftrA* ortholog paired with the *fetC* ortholog CDS0314, thus revealing the same tandem organization in *S. apiospermum*,. None of the nine putative ferric reductases identified in the *S. apiospermum* genome was located in the vicinity of these two Ftr/Fet couples.

### Transcriptional response according to iron availability

**Figure 3:**
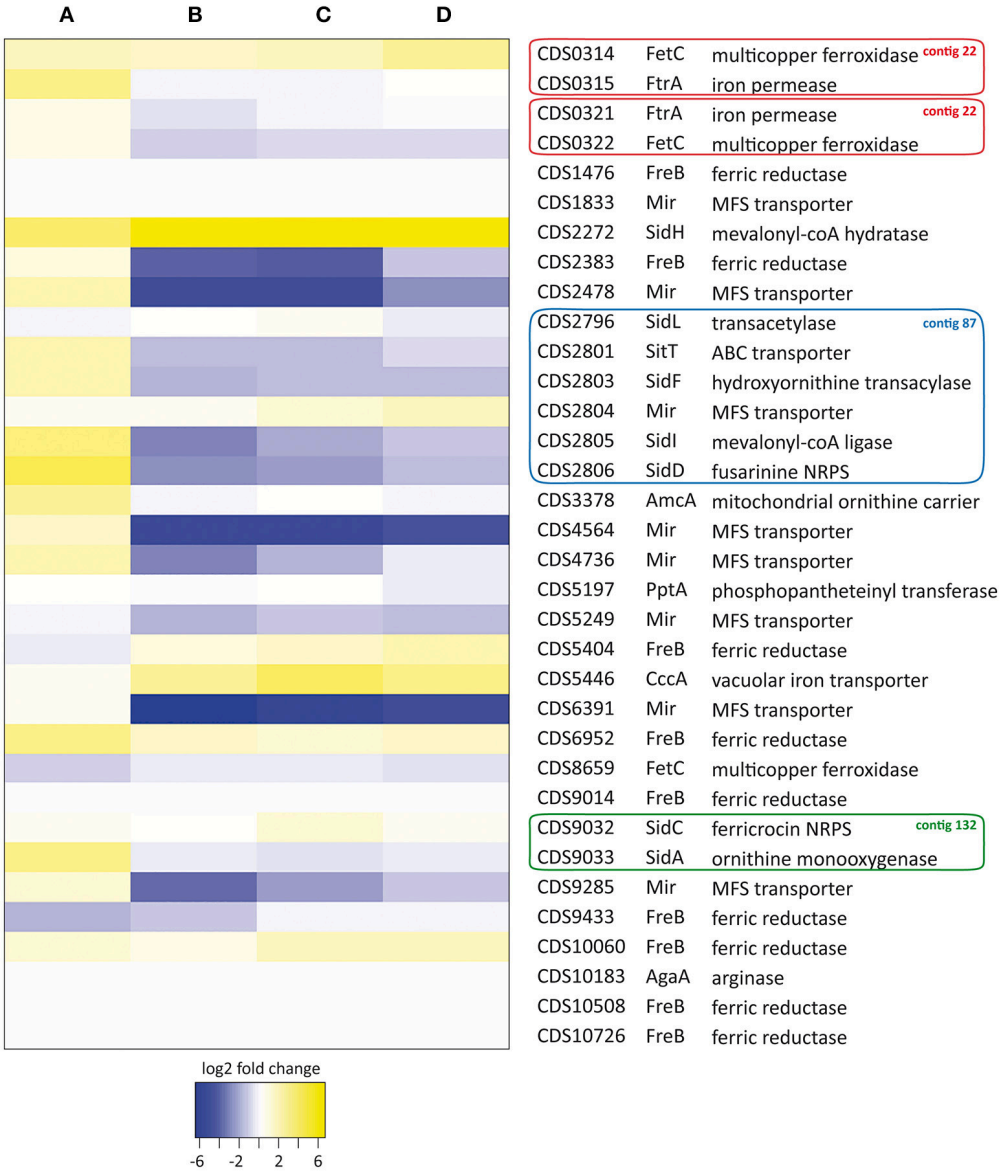
Heat map analysis of iron homeostasis genes in *S. apiospermum* IHEM 14462. Expression levels were determined by qPCR after 48 h of culture under iron starvation (**A**: 200 μM BPS) or iron excess conditions (**B**: 20 μM FeCl_3_, **C**: 20 μM FeSO_4_, **D**: 20 μM transferrin-bound iron). Relative fold-changes were log2 transformed for data analysis. Genbank accession numbers of the ORFs, as well as a summary of tBLASTn analysis, are given on the right. Besides, genes belonging to identified clusters are boxed (red: contig 22, GenBank JOWA01000022.1; blue: contig 87, GenBank JOWA01000087.1; green: contig 132, GenBank JOWA01000132.1).

To assess whether the genes predicted *in silico* were actually involved in iron homeostasis, we studied their expression in *S. apiospermum* cells grown for 48 h in iron starvation or iron excess conditions by qPCR (Figure 3). Globally, the variations of expression level observed in iron excess conditions were not influenced significantly by the source of iron (FeCl_3_, FeSO_4_ or holotransferrine).

- *Siderophore biosynthesis*. The expression of siderophore biosynthesis-encoding genes, especially those involved in extracellular siderophore production (i.e., *sidA, sidD, sidF, sidH*, and *sidI* orthologs), was highly induced under iron deprivation. By contrast, the genes involved in intracellular siderophore synthesis, i.e., *sidC* and *sidL* orthologs, as well as the putative *pptA* gene, which encodes a protein required for NRPSs activation (Allen et al., 2011), remained isoexpressed in our conditions. Besides, all of the siderophore genes that were overexpressed in iron-starved mycelia were down-regulated during iron excess, except for the *sidH* ortholog, which remained up-regulated.
- *Ornithine metabolism*. In *S. apiospermum*, the gene encoding the putative mitochondrial ornithine transporter *amcA* was up-regulated during iron starvation; by contrast, the arginase-encoding gene was barely expressed in all culture conditions tested. These results suggest that the cytosolic pool of ornithine is mainly fueled by mitochondria in *S. apiospermum*.
- *Ferrisiderophores transport*. As aforementioned, the *S. apiospermum* genome encodes a single *sitT* and 8 *mir* orthologs. In our conditions, the *sitT* ortholog and 3 out of 8 *mir* orthologs (CDS2478, 4564, and 4736) were upregulated during iron starvation. Moreover, the expression of these four loci was downregulated in iron excess conditions, strongly suggesting their involvement in siderophore-mediated iron uptake. Two other *mir* orthologs, CDS6391 and CDS9285, were strongly downregulated in iron-overloaded conditions. However, the increase in their expression level was not statistically significant during iron starvation (log2 fold-change ± standard deviation: 0.33 ± 0.50 and 1.06 ± 0.56 for CDS6391 and CDS9285, respectively).
- *Reductive iron assimilation*. Among the 9 putative ferric reductases found in the *S. apiospermum* genome, only the ortholog with the highest similarity to the *A. fumigatus freB* gene (CDS2383) was significantly down-regulated in iron-overloaded culture conditions. Four genes (CDS1476, 9014, 10508, and 10726) were not significantly expressed in any of the conditions tested, while another (CDS6952) was overexpressed under all assayed conditions. Moreover, only the RIA gene couple that displays the highest homology with the *A. fumigatus fetC/ftrA* gene cluster (CDS0314-0315) was induced under iron starvation. Besides, the ORF hypothesized to encode a laccase-type multicopper oxidase (CDS8659) was, as expected, unresponsive to all tested conditions.
- *Vacuolar iron storage*. Vacuolar sequestration probably occurs in *S. apiospermum* since the *cccA* homolog, which encodes a vacuolar iron importer in *A. fumigatus* (Gsaller et al., 2012), was significantly overexpressed during iron excess, while its expression remained unchanged under iron deprivation.

## Discussion

Iron is known to be metabolically essential for virtually all living organisms. Therefore, the “battle for iron” between a given pathogen and the host, but also between several pathogens coexisting within the same host (e.g., *A. fumigatus* and *Pseudomonas aeruginosa* in the CF lung), is a key determinant for a successful infection. To circumvent host-iron withholding, a number of bacterial and fungal pathogens have developed high-affinity iron uptake systems, some of which being mandatory for full virulence. Here, we investigated the presence of iron-related genes in *S. apiospermum* genome in order to find molecular mechanisms potentially underpinning pathogenicity.

The automated *in silico* analysis of *S. apiospermum* genome mis-annotated intronless genes as pseudogenes. Therefore the identification of genes putatively involved in iron metabolism was performed through a tBLASTn rather than a BLASTp analysis, using *A. fumigatus* Af293 iron-related proteins as query (Haas, 2012). This approach allowed to find orthologs for almost all genes involved in iron homeostasis in *A. fumigatus*. Furthermore, data mining revealed that most of the genes required for hydroxamate siderophore biosynthesis and transport were clustered in *S. apiospermum*. Such genomic organization has already been described in various filamentous fungi (Haas et al., 2008; Franken et al., 2014), but differs from one species to another (Figure 1). For instance, three distinct clusters have been identified in *A. fumigatus* and *A. nidulans* (Haas, 2012), whereas only two clusters are described in *A. niger* (Franken et al., 2014). One of these clusters contains *sidC* and *sidI* and seems to be preserved among the aforementioned three *Aspergillus* species. Interestingly, in *S. apiospermum*, the *sidC*-related cluster contains an ortholog of *sidA* instead of *sidI* (a feature also found in *T. reesei* and *C. higginsianum*), while *sidA* gene is not clustered in *Aspergillus* genomes. As for *S. apiospermum sidI* ortholog, it belongs to another cluster containing a series of genes putatively involved in siderophore biosynthesis (i.e., *sidD, sidF*, and *sidL* orthologs) and uptake (i.e., one *sitT* and one *mir* orthologs). In *Aspergillus* species, the *sidD*-related cluster also includes *sidH*, while in *S. apiospermum* the *sidH* ortholog is located in a different region of the genome together with a putative MFS transporter gene, and is separated from the *sidD* cluster by 110 kb. Moreover, like *sidA, sidL* genes are not clustered with other siderophore-biosynthetic genes in *Aspergillus* genomes. Overall, comparative genomics showed that the *S. apiospermum* siderophore genes were organized as described in most of the siderophore-producing fungi including the aspergilli. Nevertheless, gene clustering in *S. apiospermum* was more similar to that observed in phylogenetically close phytopathogenic or mycoparasitic molds such as *C. higginsianum* and *T. reesei*.

Further analysis of the two *S. apiospermum* siderophore-associated NRPS genes showed that the *sidD* ortholog encodes a protein with 44–45% sequence similarity with those produced by the three above-mentioned *Aspergillus* species. However, the precise structure of the synthesized metabolite could not be predicted on the single basis of the NRPS sequence. Indeed, although *A. fumigatus* and *A. niger* SidD are closely related (66% identity), *A. fumigatus* produces the extracellular siderophore fusarinine C, while *A. niger* synthesizes coprogen B (Franken et al., 2014). Besides, one of the closest ortholog of *A. fumigatus* SidD in non-*Aspergillus* species is found in *Metarhizium robertsii*. It displays 58% identity with *S. apiospermum* SidD ortholog and is involved in the biosynthesis of another coprogen-type siderophore termed *N*^α^-dimethyl coprogen (Giuliano Garisto Donzelli et al., 2015). We previously demonstrated that *S. apiospermum* is able to synthesize and secrete the coprogen-type siderophore *N*^α^-methyl coprogen B (Bertrand et al., 2009). HPLC-MS also evidenced that *S. apiospermum* produces the dihydroxamate dimerumic acid, but its involvement in iron metabolism is controversial since it is both described as a breakdown product of coprogen and as a natural product of several molds like *Verticillium dahliae* or *Penicillium chrysogenum* (Donzelli and Krasnoff, 2016). Together, these data suggest that *sidD* is responsible for the biosynthesis of *N*^α^-methyl coprogen B in *S. apiospermum*.

Likewise, the prediction of the final non-ribosomal peptide synthesized by *sidC* orthologs is hazardous, if not impossible. For instance, experimental studies showed that despite the high degree of similarity existing among *sidC* within the *Aspergillus* genus, the most likely intracellular siderophore produced by *A. niger* is ferrichrome (Franken et al., 2014), while *A. fumigatus* and *A. nidulans* both synthesize ferricrocin (Haas et al., 2008). Until now, only extracellular siderophores have been identified in *S. apiospermum*. Nevertheless, ferricrocin has been detected in the closely related species *S. boydii* (Vladimír Havlíček, personal communication), in which the putative *sidC* gene encodes a protein that shares 93% identity with XP_016639834.1 encoded by *S. apiospermum sidC* ortholog (CDS9032). Thus, even if mass-spectrometry analyses are needed, it is highly probable that *S. apiospermum* also produces ferricrocin.

Expression data showed that the *S. apiospermum* genes involved in extracellular siderophores biosynthesis (i.e., *sidA, sidD, sidF, sidH*, and *sidI* orthologs) were significantly induced during iron starvation. Conversely, expression of the genes specifically implicated in intracellular siderophores production, i.e., *sidC* and *sidL* orthologs, as well as the NRPS activator gene *pptA*, remained stable in this condition. The last 2 genes are known to be constitutively expressed in *A. fumigatus* (Oberegger et al., 2003; Blatzer et al., 2011c). More surprising is the unchanged expression level of the *sidC* ortholog, since previous studies based on Northern-blot analyses in *Aspergillus* showed that transcription of this gene was detectable only during iron starvation (Oberegger et al., 2002; Eisendle et al., 2003; Schrettl et al., 2007). However, more recent studies showed that *sidC* expression is only weakly affected by low iron concentrations (i.e., ≤20 μM) in both *Aspergillus* and non-*Aspergillus* species (Reiber et al., 2005; López-Berges et al., 2012; Franken et al., 2014). The iron concentration in our experimental conditions (20 μM) could be insufficient to induce significant overexpression of *sidC*. Likewise, compared to *Aspergillus* species, *S. apiospermum* is a slow-growing fungus and one can hypothesize that *sidC* overexpression is time-delayed in this species. On the other hand, all siderophore genes that were overexpressed in iron starved mycelia were down-regulated during iron excess, except for the *sidH* ortholog, which remained up-regulated. Given that the *sidH*-encoded enoyl-CoA hydratase catalyzes a reversible reaction (Abdel-Mawgoud et al., 2013), one may speculate that its overexpression in iron excess may help to drop off the peroxisomal pathway and to diminish the production of extracellular siderophores in iron-rich environments.

Among the 9 putative SIT (1 *sitT* and 8 *mir*) orthologs found in *S. apiospermum* genome, one *mir* ortholog (CDS2804) and the *sitT* ortholog (CDS2801) are part of the *sidD*-related cluster, a feature also observed in *Aspergillus* spp. and *T. reesei*. The expression of the *S. apiospermum* putative *sitT* and of 3 out of the 8 *mir* orthologs (CDS2478, CDS4564, and CDS4736) varies with iron availability, indicating that they probably participate in ferrisiderophore uptake. Strikingly, the *mir* ortholog belonging to the *sidD* cluster, as well as the four remaining genes, was not statistically overexpressed under iron depletion. In *A. fumigatus*, iron starvation induces iron-related genes transcription by the recruitment of the HapX transcription factor at CCAAT sequences present in their promoter, through the interaction of HapX with the CCAAT-binding complex (CBC) (Hortschansky et al., 2007). Promoter analysis of four SIT encoding genes responsive to iron starvation (CDS2801, 2478, 4564, 4736) revealed the presence of 3 to 5 CCAAT motifs while only one CCAAT motif was found in the promoter of the unresponsive transporter gene CDS2804 (Supplementary Table S2). Thus, we first hypothesized that the level of expression of SIT genes could be related to the number of CCAAT motifs present in their promoter. Nevertheless, we also found 2 to 5 CCAAT motifs in the promoter of *mir* orthologs unresponsive to iron starvation (CDS1833, 5249, 6391, and 9285). Of note, 3 out of these 4 Mir-encoding genes (CDS1833, 6391, and 9285) belong to gene clusters also organized around some NRPS encoding genes (CDS1828, 6390 and 9291, respectively), but totally unrelated to iron metabolism (unpublished results). The absence of induction of the *mir* orthologs CDS2804 and 5249 remains to be explained.

Despite an apparent expansion of the gene set putatively involved in RIA (*n* = 14), only three (CDS2383, 0314, and 0315) showed adequate response to the tested conditions, i.e., up-regulation during iron starvation and/or down-regulation during iron excess. RIA is a tripartite system made of one metalloreductase associated with a ferroxidase/ferripermease tandem. *Aspergillus fumigatus* genome harbors 15 putative metalloreductase genes, but only one, namely *freB*, is involved in iron metabolism (Blatzer et al., 2011b). Transcriptional analysis showed that the expression of *freB* was repressed by the GATA transcription factor SreA during iron sufficiency. Interestingly, the only putative *S. apiospermum* ferric-reductase gene that was down-regulated by iron corresponded to the best hit with the *A. fumigatus* ortholog (CDS2383). Likewise, only the RIA gene cluster showing the highest degree of homology with the *A. fumigatus fetC/ftrA* gene pair was significantly overexpressed during iron starvation (CDS0314/CDS0315). Of note, two FetC/FtrA homologs with distinct functions are described in the yeast *Saccharomyces cerevisiae*; indeed the Fet3p/Ftr1p complex mediates Fe(III) channeling in the yeast plasma membrane, while the paralog Fet5p/Fth1p complex mediates iron moves from the yeast vacuole (Urbanowski and Piper, 1999). Consequently, one may hypothesize that the non-responsive *S. apiospermum fetC/ftrA* gene cluster is involved in vacuolar trafficking of iron rather than in RIA. Moreover, we could identify an iron transporter *cccA* ortholog in the genome, the expression of which was significantly induced in iron-overloaded cells, suggesting that a vacuolar iron homeostasis system exists in *S. apiospermum*.

Altogether, these findings indicate that *S. apiospermum* possesses genetic information needed for iron uptake and regulation. Expression data suggest that, in mycelia, iron acquisition is mediated by both RIA and the siderophore system. Our research group already evidenced the production of extracellular siderophore in *S. apiospermum*, and our genomic analysis found putative orthologous genes for both extra- and intracellular siderophore biosynthesis. Works are in progress to identify all the hydroxamate-type siderophores produced by *S. apiospermum*, with a particular emphasis on intracellular siderophore biosynthesis. Moreover, the role of siderophores during *Scedosporium* infections has not been studied so far, and experiments in a rodent model of scedosporiosis are planned to evaluate their implication in *Scedosporium* virulence.

## Author contributions

YL, J-PB, PV: Conceived and designed the experiments; YL, PV: Performed the experiments; YL, NP, SL, BL, PV: Analyzed the data; YL, PV: Wrote the paper. All authors read and approved the manuscript.

### Conflict of interest statement

The authors declare that the research was conducted in the absence of any commercial or financial relationships that could be construed as a potential conflict of interest.
