# Supplementary material for: Genomic Organization and Expression of Iron Metabolism Genes in the Emerging Pathogenic Mold Scedosporium apiospermum
Source: Front Microbiol. 2018 Apr 26;9:827. doi: 10.3389/fmicb.2018.00827 (PMC5932178; doi:10.3389/fmicb.2018.00827)
Supplement: Supplementary file 1 [file Table_1.DOCX]

**Supplementary Table S1:** List of primers used in this study

| Primers | Sequence | Use |
| --- | --- | --- |
| Sa9033-F Sa9033-R | 5’- CACACCTCAACTTGCTCAAG -3’ 5’- GAGTTTCCTAGTCTGCGACA-3’ | qPCR qPCR |
| Sa9032-F Sa9032-R | 5’- CCAGGTCCTGGATGATTCTTC-3’ 5’- GATGCCAATATCTAGCCCGC-3’ | qPCR qPCR |
| Sa2806-F Sa2806-R | 5’- CAGTTGTGCACCAGGATGTC-3’ 5’- CGACTTCCTTCCAAGACTCG-3’ | qPCR qPCR |
| Sa2803-F Sa2803-R | 5’- CAGGTACCGAGGACCTCATA-3’ 5’- CAAACGCACTCCTCTTGTGA-3’ | qPCR qPCR |
| Sa2272-F Sa2272-R | 5’- GTAGCCAGATCTTCACGACG-3’ 5’- GATAACCATCTCCATGCCGC-3’ | qPCR qPCR |
| Sa2805-F Sa2805-R | 5’- CACCAAGTCTGCAACCTCCA-3’ 5’- GCATGCGGTCACCAATGAAG-3’ | qPCR qPCR |
| Sa2796-F Sa2796-R Sa2796bis-F Sa2796bis-R Sa2796-F-start Sa2796-R-stop | 5’- GAGGCAGCGGATATGAAGAC-3’ 5’- CTAAGACCGGCGAAGGAATG-3’ 5’- GACTTGGCTGTCGAGCTTGG -3’ 5’- CTCGAGAATCCCGCCTTCTG -3’ 5’- GGCGCCCCAGGTCATCAAC -3’ 5’- CACGACTCCAAGTCCCCTC-3’ | PCR PCR qPCR/PCR qPCR/PCR PCR PCR |
| Sa5197-F Sa5197-R | 5’- CGAGGGACCTTTTCCCTGAAG -3’ 5’- CTAGAGAGAGCTTGGCGTCTC -3’ | qPCR qPCR |
| Sa10183-F Sa10183-R | 5’- ACCAACCGAGACTGTTAGGG-3’ 5’- CGTCCAGATAGCTTCGATG-3’ | qPCR qPCR |
| Sa3378-F Sa3378-R | 5’- GTGCCCTCGAAGCTGTTGAA-3’ 5’- TTGTGAGGCTGCGACTGAAG-3’ | qPCR qPCR |
| Sa2478-F Sa2478-R | 5’- GTGGCAGGACGCTACTTCAA-3’ 5’- GCCAATACTGCGAATCCGAC-3’ | qPCR qPCR |
| Sa2804-F Sa2804-R | 5’- GGAGTATTTGCATCGGTTGG-3’ 5’- GACTTCTGCTTTCCGAGGTC-3’ | qPCR qPCR |
| Sa1833-F Sa1833-R | 5’- GTCGAGAGCTACCAGCATGT-3’ 5’- CCACCTTGTGAACCTCAACC-3’ | qPCR qPCR |
| Sa4564-F Sa4564-R | 5’- GAGAACTCTGACGCCCAAAG-3’ 5’- CCGAACGAAGACGTCACGAA-3’ | qPCR qPCR |
| Sa4736-F Sa4736-R | 5’- GCTCATGACCAGAGTATCAGC-3’ 5’- GCTTGCGGAAGTGAATGAGG-3’ | qPCR qPCR |
| Sa5249-F Sa5249-R | 5’- GCGCATCCTTACCATCATTG-3’ 5’- ACGGAATATCACGCCTCTCA-3’ | qPCR qPCR |
| Sa6391-F Sa6391-R | 5’- CAAGACTCTCGTCGGTGTGG-3’ 5’- GATTGGGCCTTGTCTTTGGC-3’ | qPCR qPCR |
| Sa9285-F Sa9285-R | 5’- GGCGAGACTTCGGATACTTC-3’ 5’- GATGCCAGACGAGAAAGCGT-3’ | qPCR qPCR |
| Sa2801-F Sa2801-R | 5’- GGTCAAATCTACACCGTGGT-3’ 5’- TACCGTTGATAGCAGGTTGG-3’ | qPCR qPCR |
| Sa5446-F Sa5446-R | 5’- GGACACCAAGGTCGTTAT-3’ 5’- GTCAGAATGGACGGAAAC-3’ | qPCR qPCR |
| Sa0314-F Sa0314-R | 5’- CGCTGCTGCAAATACCCAA -3’ 5’- CCTTCTTCAAATCCAGCGC -3’ | qPCR qPCR |
| Sa0315-F Sa0315-R | 5’- GATCTCCACAGGAGCGTCT -3’ 5’- GGATAGTGCTGATAGTGGC -3’ | qPCR qPCR |
| Sa0321-F Sa0321-R | 5’- GAAGCAGACTCTAGACCGG -3’ 5’- CGAACCCGTAGAAAATACCG -3’ | qPCR qPCR |
| Sa0322-F Sa0322-R | 5’- CGCTGCTGGAAACACTCATA -3’ 5’- CAATGCCCATGATGCAGCTG -3’ | qPCR qPCR |
| Sa8659-F Sa8659-R | 5’- GCGGGAGGAAATGTTGAGG -3’ 5’- GATACGCGAGCGCTTCACAC -3’ | qPCR qPCR |
| Sa1476-F Sa1476-R | 5’- GAGGATGACGCTTACTGGAG -3’ 5’- CGATCACGTAGGCGTAGAAG -3’ | qPCR qPCR |
| Sa2383-F Sa2383-R | 5’- GCGTCGATGAGGAACAATCT -3’ 5’- CGACTATCGATGCGCGAACT -3’ | qPCR qPCR |
| Sa5404-F Sa5404-R | 5’- CGGTCGTATCGTCATCATCG -3’ 5’- CGTAGAAAGCGTGTCGGAC -3’ | qPCR qPCR |
| Sa6952-F Sa6952-R | 5’- GCGCCAGAACTAGACATTCT -3’ 5’- GCCGATCATGTTAGCGTCAA -3’ | qPCR qPCR |
| Sa9014-F Sa9014-R | 5’- CTCTGGAACAAGTCACTACGC -3’ 5’- GAAGTGTGATGGCAATGGGAG -3’ | qPCR qPCR |
| Sa9433-F Sa9433-R | 5’- CGCGCGGATACTTTACAATC -3’ 5’- CAGGAGGAACTGCGTCATGT-3’ | qPCR qPCR |
| Sa10060-F Sa10060-R | 5’- CCGAAAGGACATTCTCCGTA-3’ 5’- GCAAACAGTGACGCAGAGAG-3’ | qPCR qPCR |
| Sa10508-F Sa10508-R | 5’- CCCGCAAGCCGTTGAATGTT-3’ 5’- GATGGGAAGGAGGATGGCAA-3’ | qPCR qPCR |
| Sa10726-F Sa10726-R | 5’- CGAATCGTGATGCTTGGCGG-3’ 5’- GGAGCTCTTGAATGTGGTCG-3’ | qPCR qPCR |
| SaUbcB-F* SaUbcB-R* | 5’- CCGGAGAGTTGTGCCTTGA-3’ 5’- CAGGTTCGATGCTTCGACG-3’ | qPCR qPCR |
| SaSarA-F* SaSarA-R* | 5’- CATCAGCTCGGTCTCTACCA-3’ 5’- CGTATTGGGACAACCACCGT-3’ | qPCR qPCR |

*Reference genes used for normalization of gene expression data.
